# Prognostic factors in patients with node-negative gastric cancer: an Indian experience

**DOI:** 10.1186/1477-7819-9-48

**Published:** 2011-05-10

**Authors:** Ramakrishnan A Seshadri, Sunil B Jayanand, Rama Ranganathan

**Affiliations:** 1 Department of Surgical Oncology, Cancer Institute (WIA), Chennai-600036, India; 2 Department of Biostatistics and Tumor Registry, Cancer Institute (WIA), Chennai-600036, India

## Abstract

**Background:**

The status of the regional nodes is the most important prognostic factor in gastric cancer. There are subgroups of patients with different prognosis even in node-negative patients of gastric cancer. The aim of this study is to analyze the factors influencing the prognosis in Indian patients with node-negative gastric cancer.

**Methods:**

This was a retrospective analysis of patients who underwent radical gastrectomy in a tertiary cancer centre in India between1991 and 2007. The study group included only patients with histologically node-negative disease. Various clinical, pathological and treatment related factors in this group of patients were analyzed to determine their prognostic ability by univariate and multivariate analyses.

**Results:**

Among the 417 patients who underwent gastrectomy during this period, 122 patients had node-negative disease. A major proportion of the patients had advanced gastric cancer. The 5-year overall survival and disease-free survival in all node-negative gastric cancer patients was 68.2% and 67.5% respectively. The overall recurrence rate in this group was 27.3%. On univariate analysis, the factors found to significantly influence the disease-free survival were the size, location and presence or absence of serosal invasion of the primary tumor. However, on multivariate analysis, only tumor size more than 3 cm and serosal invasion were found to be independently associated with an inferior survival.

**Conclusion:**

Serosal invasion and primary tumor size more than 3 cm independently predict a poor outcome in patients with node-negative gastric cancer.

## Background

The status of the regional lymph nodes is the most important prognostic factor in gastric cancer and patients with node-negative gastric cancer have a better survival compared to those with nodal metastasis [1,2]. However, even among the node-negative patients, there are certain subgroups of patients who fare better than the others [3-13]. Identification of the poor risk category among the node-negative patients may help in planning adjuvant therapy for this group. Although the incidence of gastric cancer in India is less than other Asian countries like Japan, Korea and China, the age-standardized (world) incidence of gastric cancer in Chennai (12.2 and 6.0/100,000 population in males and females respectively) is among the highest in India [14]. While many previous studies on node-negative gastric cancer have included a large proportion of patients with early gastric cancer, we wished to study the factors influencing the survival in Indian patients with node-negative gastric cancer, most of whom have advanced gastric cancer. This will help in the optimal utilization of treatment resources for better patient care.

## Methods

We undertook a retrospective analysis of patients diagnosed with gastric cancer who underwent radical gastrectomy with a curative intent in our institution between 1991 and 2007. Prior to 1993, most patients underwent a D1 dissection. From 1993, D2 dissection was the standard treatment for patients with gastric cancer and a D1 dissection was performed only in special situations (patients above 70 years, severely malnourished patients or the occasional patient who underwent emergency surgery for bleeding from the tumor). Only patients in whom routine histological examination (hematoxylin-eosin staining) of the dissected regional lymph nodes did not reveal evidence of nodal metastasis were included in the study group. The UICC TNM 6^th ^edition staging system was used for staging the disease [15]. Patients in whom all examined lymph nodes were negative for metastasis regardless of the number of nodes dissected were still designated as pN0 according to the UICC TNM guidelines [15] and were included in the analysis. Patients with carcinoma-in-situ were excluded from the study, since the risk of nodal metastasis is very low in these patients and they cannot be compared with invasive malignancies. 

Adjuvant chemotherapy was not administered to any of the patients. The tumors were divided into proximal or distal by an imaginary line between the incisura and the mid-point of the greater curve. The patients were followed 3-monthly for the first three years, 6-monthly for the next two years and then annually thereafter. Clinical examination was performed at each visit and abdominal imaging studies (computerized tomography scan or an ultrasound) was requested every year for the first five years. An endoscopy was performed once a year for three years. Local recurrence identified on endoscopy was always confirmed by a biopsy. Nodal or distant recurrences were identified on imaging and a biopsy was not attempted unless the imaging result was equivocal. The median duration of follow-up of all patients in this study (including 5 patients who had a 30-day mortality) was 58 months (range 1 to 202 months). Univariate analysis of various prognostic factors influencing the disease-free survival (DFS) was performed using log-rank test. Factors identified to be significant in univariate analysis were included in the multivariate analysis, which was performed using the Cox proportional hazard model. The survival estimates were calculated by life-table method. Statistical significance was considered when *p *value was < 0.05. The SPSS v11.0.1 software was used for statistical analysis

## Results

Among the 417 patients who underwent radical gastrectomy during the study period, 122 patients had histologically node-negative disease. After excluding one patient with carcinoma-in-situ, the study population had 121 patients (29%). Table 1 enlists the patient, surgical and pathological details of the node-negative patients. More than 80% of the patients had advanced gastric cancer (predominantly T3 disease) and D2 lymphadenectomy was performed in 81% of the patients.

**Table 1:** Clinical, pathological and treatment details of node negative patients

Variable	No. (%)
Gender	
Male	86
Female	35

Median age (range)	53 years (27-76)

Depth of invasion	
pT1	22 (18.2)
pT2	33 (27.3)
pT3	64 (52.9)
pT4	2 (1.7)

Size of primary tumor	
≤3 cm	18 (14.9)
>3 cm	85 (70.2)

Grade	
1	12 (9.9)
2	39 (32)
3	70 (57.9)

Histology	
Adenocarcinoma	102 (84.3)
Mucin secreting carcinoma	13 (10.7)
Signet-ring cell carcinoma	6 (5)

Type of gastrectomy	
Distal	97 (80.2)
Total	21 (17.4)
Proximal	3 (2.5)

Extent of lymphadenectomy	
D1	23 (19)
D2	98 (81)

Median no. of dissected nodes (range)	22 (4-77)

No. of nodes dissected	
≤15	39 (32.2)
>15	82 (67.8)

On follow-up, recurrence was detected in 33 patients (27.3%). Of this, distant metastasis was seen in 24 patients (19.8%), isolated locoregional recurrence in 6 patients (4.9%) and combined locoregional and distant recurrence was seen in 3 patients (2.4%). The 3-year and 5-year overall survival in node-negative patients were 76% and 68.2% respectively, whereas the 3-year and 5-year disease-free survival were 73.6% and 67.5% respectively. In contrast, the 5-year overall survival of the 295 patients who had node-positive disease was 29.1%, which was significantly lower than that of node-negative patients (*p *< 0.001).

**Figure 1:**
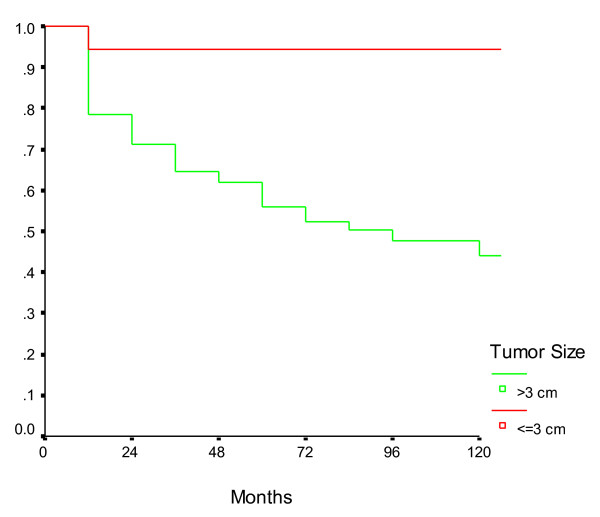
**Survival in node negative gastric cancer patients according to tumor size**. The disease-free survival of patients with tumor size ≤3 cm was higher than that of patients with tumors size >3 cm.

**Figure 2:**
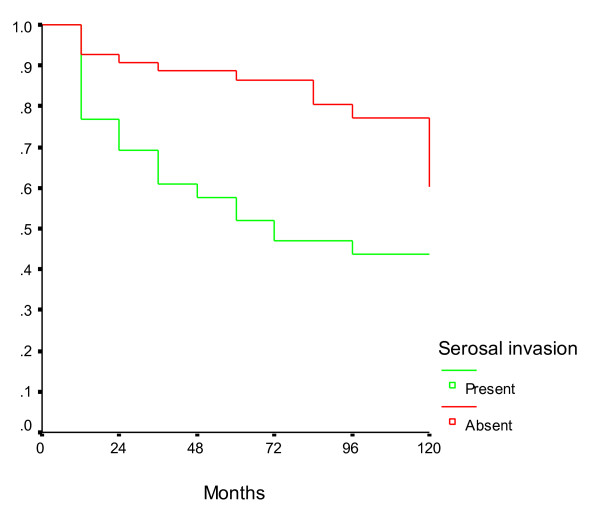
**Survival in node negative gastric cancer patients according to serosal invasion**. The disease-free survival of patients with serosal invasion was lower than that of patients without serosal invasion.

On univariate analysis, the only factors which were found to be significantly associated with a poor disease-free survival in patients with node-negative gastric cancer were tumor size >3 cm, proximal tumor location and the presence of serosal invasion in the primary tumor (Table 2). The age of the patient, gender, histology and grade of the tumor did not correlate significantly with survival. There was a non-significant trend towards better survival in patients in whom >15 nodes were dissected when compared to those with fewer number of dissected nodes. Similarly, although there was a trend towards improved survival in patients undergoing D2 lymphadenectomy when compared to D1, we could not demonstrate any statistical significance. On multivariate analysis, only the primary tumor size >3 cm and presence of serosal invasion were found to be independently associated with an inferior disease-free survival (Table 3). The survival curves in patients with node-negative gastric cancers according to tumor size and serosal invasion are presented in Figures 1 and 2 respectively.

**Table 2:** Univariate analysis of factors predicting disease-free survival in node negative gastric cancer

Variable	5-year Disease free survival	p value
1. Gender		
Male	68.2%	0.78(NS)
Female	65.4%	

2. Age		
≤53 years	63.5%	0.557(NS)
>53 years	71.5%	

3. Haemoglobin		
≤9 gm%	59.03%	0.42(NS)
>9 gm%	70.6%	

4. Patient Weight		
≤50 kg	68.7%	0.44(NS)
>50 kg	66.5%	

5. Location of tumor		
Distal	71.8%	**0.05**
Proximal	48.6%	

6. Multi-organ resection		
None	68.2%	0.69(NS)
Pancreaticosplenectomy	55.4%	
Others	100%	

7. Dissection		
D1	53.5%	0.100(NS)
D2	70.1%	

8. Histology		
Adenocarcinoma	65.5%	0.30(NS)
Mucinous carcinoma	84.6%	
Signet ring cell carcinoma	62.5%	

9. Grade		
1	80%	0.14(NS)
2	76.8%	
3	60%	

10. Tumor size		
≤3 cm	94.3%	**0.003**
>3 cm	55.9%	

11. Serosal invasion		
Absent	86.4%	**0.0002**
Present	51.9%	

12. No of nodes dissected		
≤15	59.6%	0.07(NS)
>15	71.3%	

13. Gastric outlet obstruction		
Absent	70.3%	0.27(NS)
Present	60.7%	

14. Blood loss		
≤400 ml	72.9%	0.17(NS)
>400 ml	64.3%	

NS- not significant

**Table 3:** Multivariate analysis of prognostic factors in node negative gastric cancer

Variable	Hazard ratio	95% confidence interval	p value
1. Location of tumor			
Distal	1*		
Proximal	1.43	0.73-2.79	0.36(NS)

2. Tumor size			
≤3 cm	1*		
>3 cm	7.97	1.08-59.06	**0.04**

3. Serosal invasion			
Absent	1*		
Present	2.13	1.04-4.35	**0.04**

*Reference value NS- not significant

## Discussion

The status of regional nodes is the most important prognostic factor in gastric cancer [1,2]. Node-negative patients have a significantly better survival when compared to node-positive patients. The 5-year and 10-year overall survival in node-negative patients have been reported to vary from 72% to 91.7% [2-10] and 88% to 93% [3,6,11] respectively. The recurrence rates vary from 13.7% to 29.4% in this favorable subset of patients [7,8,10,12]. Most of these series come from East Asian countries and include a sizeable proportion of early gastric cancer. Our series, which reflects the Indian scenario, has a majority of patients diagnosed with advanced gastric cancer and hence the 5-year survival rates reported by us (68.2%) is slightly less than that quoted in the literature. However, in one of the largest series, comprising more than 1500 patients with node negative gastric cancer, Kim *et al *[13] reported a comparable 5-year survival (66.9%) in patients with advanced gastric cancer.

The proportion of node-negative patients in our study, although less than that reported from many East Asian countries [5,11,13], is comparable to that reported in some western centers [4,9]. There are two reasons for this. First, aggressive screening programmes, as practiced in Japan leads to early detection of gastric cancer whereas in India, many patients present in an advanced stage due to the lack of such screening programmes. Second, it has been observed that node-negative gastric cancer is frequently associated with small tumor size (usually <4 cm) [3,5,11], less poorly differentiated tumors [3] and are usually confined to the muscularis propria [3,11]. In the entire population of 417 gastric cancer patients treated in our institution, the mean size of the primary tumor was 5.3 cm, 69% of the patients had high grade tumors and 75% of the patients had disease extending beyond the muscularis propria (data not shown) - all of which probably account for a high proportion of nodal metastasis among Indian patients.

Various clinico-pathological factors have been reported to influence the survival and recurrence rates in node-negative gastric cancer. The size of the primary tumor has been reported to be a significant prognostic factor for survival in earlier studies [5,6,10,13,16], and has been confirmed in our study as well. The presence of serosal invasion, which emerged as an independent prognostic factor in our study has been documented by other authors [4,8,10,12,13,16]. The depth of invasion of the primary tumor has also been reported as a prognostic factor [3,11,17-19]. Proximal location of the tumor was significant only on univariate analysis in our study, but lost significance on multivariate analysis. Similarly, distal tumor location did not emerge as an independent prognostic factor in other studies [3,19]. In contrast, Deng *et al. *[8] found that patients who underwent subtotal gastrectomy had a significantly longer median overall survival when compared to those who underwent a total gastrectomy (116 vs 91 months, p = 0.03), which indirectly reflected the influence of tumor location on survival. Lauren's histological classification has been reported to be an independent prognostic factor in node-negative gastric cancer, with the intestinal type having a better prognosis than the diffuse type [12,19]. Even though we did not use the Lauren's classification in our study, the histological subtype of the tumor according to the WHO classification did not emerge as a significant prognostic factor in our analysis. The other factors which have been found to influence survival in patients with node-negative gastric cancer include presence of lympho-vascular invasion [3,12,16,18,20], age [11,17,18,21], gender [8], vascular invasion [5,22], S-phase fraction [23] and proliferating cell nuclear antigen (PCNA) labeling index [24]. Poorly differentiated tumors also have a poor prognosis [4,10], although in our analysis we were not able to demonstrate a significant association between the histological grade of the tumor (which denotes the tumor differentiation) and survival.

Extended lymphadenectomy has been reported to be an independent prognostic factor for survival in node-negative gastric cancer by few authors [6,21,25] whereas others have reported that this survival benefit is limited only to patients withT3 disease [9,17]. It is recommended that a minimum of 15 nodes be dissected for proper staging in gastric cancer [15]. The number of node to be dissected in patients with node-negative gastric cancer is unclear, although Giuliani et al recommended examination of at least 23 nodes in these patients for identifying prognostic indicators [26]. Huang *et al. *[7] found that the number of dissected lymph nodes was an independent prognostic factor for survival in patients with node-negative gastric cancer and that there was a negative correlation between the number of dissected nodes and recurrence rates. By cut-point analysis, these authors reported better survival in pT1-2 patients in whom ≥15 nodes were dissected, in pT3-4 patients in whom ≥20 nodes were dissected and in the entire group of patients if ≥15 nodes were dissected. In a retrospective analysis, Biffi et al reported that the disease-free and overall survival of node-negative gastric cancer patients was significantly improved when at least 15 nodes were dissected regardless of the pT stage and that the risk of distant metastasis decreased with increase in the number of dissected nodes [27]. Shen et al. [28] reported that there was a positive association between the number of nodes dissected and the chance of identifying nodal metastasis in pT3 but not pT1 or pT2 gastric cancer (HR = 1.014, 95% CI 1.006-1.021). However, they failed to demonstrate an independent prognostic value of the number of nodes dissected in pT3N0 patients. In another study [8], it was observed that patients in whom more than 20 nodes were dissected had a longer median disease-free survival than patients in whom fewer than 20 nodes were dissected (47.5 versus 21.4 months, p = 0.01). In our study, although there was a definite trend towards better survival in patients who underwent D2 dissection and in patients in whom >15 nodes were examined, we were not able to demonstrate a statistically significant association between these factors and patient survival. This is probably due to the small sample size.

The benefit of extended lymphadenectomy seems to be due to the eradication of potential micrometastatic disease [7]. Using reverse transcription polymerase chain reaction assay, Wu *et al. *[29] detected micrometastasis in 20% of patients who were determined to be node negative on routine hematoxylin-eosin stains. Immunohistochemical studies have also been used to detect micrometastasis in 10-32% patients with gastric cancer [30-33]. The presence of micrometastasis significantly impacts the prognosis of patients with node-negative gastric cancer. Patients who do not harbour micrometastasis have been found to have a significantly better survival than those who harbour micrometastasis in the nodes [29,31-35]. The type of micrometastasis also seems to have an impact on the outcome. Yasuda *et al*. observed that patients with ≥4 micrometastasis have a significantly worse outcome when compared to those with fewer micrometastasis and also that those with micrometastasis in extragastric nodes fare poorly [32]. In another study, Cao *et al *reported that the cluster-type micrometastasis had a significantly poor survival when compared to the single-cell type micrometastasis [31]. The presence of micrometastasis in the nodes has been correlated with loss of E-cadherin expression in the primary tumor as well as the size, depth of invasion and differentiation of the primary tumor [30-35]. It is interesting to note that most of the variables are themselves independent prognostic factors in node-negative gastric cancer, and therefore, their prognostic importance may be attributed to the presence of micrometastasis. Thus, identifying micrometastasis in node-negative gastric cancer patients may help in prognostication as well as determining the need for adjuvant therapy.

The prognostic value of adjuvant therapy in node-negative gastric cancer has not been reported in the English literature. Adjuvant chemotherapy has been shown to improve the survival in gastric cancer in general [36,37]. However, it is difficult to say whether adjuvant chemotherapy will add to the already favourable prognosis of node-negative gastric cancer. This can be answered only by a multicenter randomized trial of adjuvant chemotherapy exclusively in patients with node-negative gastric cancer.

## Conclusion

Although patients with node-negative gastric cancers had a favorable survival in our study, the subgroup of patients with a tumor size more than 3 cm or tumors invading the serosa had a worse prognosis when compared to those having smaller tumors or those without serosal invasion. The low rate of locoregional recurrence in our study may be related to the extended nodal dissection performed in most patients. An attempt should be made in all node-negative patients to identify lymph node micrometastasis since it may further help in prognostication. The available evidence from literature suggests that an extended lymphadenectomy with dissection of at least 15 nodes must be performed even in patients with clinically negative nodes. However, surgical treatment alone is unlikely to prevent distant recurrences, even in node-negative gastric cancer. Hence, systemic therapy may have a role in node-negative gastric cancer patients. In a country like India, where it is essential to utilize the available treatment resources judiciously, patients with node-negative gastric cancer with a tumor >3 cm or invading the serosa or with lymph node micrometastasis should be considered for more aggressive adjuvant therapy in the form of systemic chemotherapy.

## Competing interests

The authors declare that they have no competing interests.

## Authors' contributions

RAS was involved in the conception and design of the study, extraction of data, interpretation of data, literature search and critical appraisal and revision of the manuscript. SBJ was involved in extraction of data, statistical analysis, literature search and writing the first draft of the manuscript. RR was involved in statistical analysis, interpretation of data and review of the manuscript. All authors read and approved the final manuscript.
